# Systematic Investigation on the Structure-Property Relationship in Isotactic Polypropylene Films Processed via Cast Film Extrusion

**DOI:** 10.3390/polym12081636

**Published:** 2020-07-23

**Authors:** Federico Di Sacco, Markus Gahleitner, Jingbo Wang, Giuseppe Portale

**Affiliations:** 1 Macromolecular Chemistry and New Polymeric Materials, Zernike Institute for Advanced Materials, University of Groningen, Nijenborgh 4, 9747 AG Groningen, The Netherlands; f.di.sacco@rug.nl; 2 Dutch Polymer Institute, P.O. Box 902, 5600 AX Eindhoven, The Netherlands; 3 Borealis AG, St. Peterstr. 25, A-4021 Linz, Austria; Markus.Gahleitner@borealisgroup.com (M.G.); Jingbo.Wang@borealisgroup.com (J.W.)

**Keywords:** cast film extrusion, chill-roll temperature, polypropylene, processing, transparency

## Abstract

The effect of cast film extrusion processing conditions, such as the chill-roll temperature, temperature of the melt, and line speed, on the structure of different isotactic polypropylene homo- and random copolymers has been investigated by means of Small- and Wide-Angle X-ray Scattering (SAXS and WAXS) and correlated to stiffness and haze. Stiffness and transparency have been found to be strongly dependent on the temperature of the chill-roll. Interestingly, line speed has been found to affect the total crystallinity when the chill-roll temperature is increased, while an overall minor effect of the melt temperature was found for all cast films. The polymer characteristics, defined by the catalyst nature and comonomer content, affect the final material performance, with the single-site catalyzed grades performing better in both mechanics and optics. Haze levels were found to correlate with the mesophase content rather than to α-crystallinity and to be dependent on the domain size for all grades. The remarkably low haze levels reached by the single-site grade with higher isotacticity can arise from high nucleation rate and orientational effects, which ultimately yield smaller and smoother scattering domains.

## 1. Introduction

Isotactic polypropylene (iPP) has encountered a continuous production growth since its market appearance in the mid-1950s [1]. It is the high versatility of this semi-crystalline polymer, in terms of both properties modification and processing design, that allows a wide range of applications from commodities to higher-end products [2,3]. Use of polypropylene as packaging material in medical applications is growing steadily every year thanks to its high transparency, toughness, and great resistance to sterilization procedures [4]. In this application sector, cast film extrusion technology allows the production of film sheets of various chemical compositions and thicknesses. Here, the polymer melt is extruded from a slit die onto a relatively cold roll. This so-called chill-roll is usually water-cooled or heated to a temperature in the range of 15 °C to 80 °C to ensure a rapid cooling. The chill-roll is then followed by further take-off and other rolls to refine and control the sheet dimension. With this technology, and variations like roll-stack technology, it is possible to cover a thickness range of 0.05–3.0 mm with a limited degree of orientation. Here, mostly homopolymers and random copolymers with ethylene, with a melt flow rate (MFR) around 8–16 g/10 min, are selected, and the final material properties can be strongly influenced by the processing condition used [5]. Melt temperature, wind-up line speed, and extrusion die distance, together with the chill-roll temperature, are fundamental parameters that can be modified to tune the final materials’ properties. 

Next to the machine setup, the variation of the iPP chain structure in terms of regio- and stereo-regularity, as well as the presence of comonomer units, has a strong effect on the final cast film products. Control over the regularity is achieved by a selection of the catalyst type. Most iPP is nowadays produced via a Ziegler-Natta catalyst (ZNC), a minor part with metallocene or single-site catalysts (SSC), resulting in major differences in terms of chain stereostructure [6]. Novel single-site catalysts allow polymerization of olefins to polymers with narrow molecular weight distribution and excellent stereo-regularity. Moreover, different stereospecific structures, such as atactic, syndiotactic, and more are accessible via metallocene catalyst. A drawback of SSC-iPP grades is the consistent presence of regio-defects, arising from the mis-insertion of monomeric units, which ultimately reduce the crystallinity and the mechanical stiffness. ZNC-iPP presents higher polydispersity compared to any SSC grades, with a value above 5, and this is often an advantage during crystallization under industrial conditions thanks to the higher crystal orientation caused by the higher molecular weight fractions [7]. Narrowed molecular weight distribution has slower crystallization kinetics and usually yield material with slightly lower crystallinity, this being an advantage in cast film technology where transparency and low stiffness are required. 

Optical and mechanical performance can be affected by modification of the chain regio- and stereo-regularity [8,9], through the introduction of comonomers like ethylene [10] or 1-butene [11], via the addition of nucleating agents [12,13] and by controlling the crystallization behavior [2,3,14]. To achieve superior optical properties, a fine control on the iPP polymorphic behavior is needed. Three types of polymorphs can be obtained (α, β, γ) as functions of the crystallization conditions, [15,16] plus a solid, highly-disordered phase called mesophase [17]. While the most common α-monoclinic phase-yield stiff material with high crystallinity, mesophase-based materials possess remarkable ductility, easier processability and, thanks to its nodular morphology, better transparency. Mesophase forms in the condition of high supercooling of the melt, at rates higher than 100°K/s. Inhibition of crystalline formation can be achieved by quenching at a rate of 1000°K/s, below the glass transition temperature [17,18]. 

In recent years, great focus has been put on cast film extrusion for producing polyolefinic film materials with remarkable mechanical and optical performance. Due to the strong cooling rate during this processing, investigation of the mesophase formation has been closely linked to cast film technology. A first study by Lamberti et al. tested the effect of the die distance on the orientation and crystalline dimensions in polypropylene cast film [19]. More recently, the effect on the optical performance by inclusion of additives such as slip and anti-blocking agents as well as acid scavengers, has been found to increase the film surface roughness and thus worsening the transparency [12,13,20]. Simulation on the cast film process by Zhou at el, found the draw-ratio to strongly effect the morphological evolution in iPP cast film [21]. Chill-roll temperature has been found to largely affect the crystallization mechanism of iPP, yielding mostly the mesomorphic phase at low chill-roll temperatures and highly transparent films, and monoclinic alpha phase products at high roll temperatures, while a minor contribution to the crystallinity was found for both melt temperature and line speed [9,22]. 

In this manuscript, a clear correlation between the cast film extrusion processing parameters on one side and the optical and mechanical properties on the other side is presented for iPP grades ranging from different homopolymers to a random copolymer produced by different catalytic routes. The link between processing and properties is possible via the detailed structural analysis conducted by wide- and small-angle X-ray scattering. The combined effect of polymer structural composition and cast film extrusion machine settings have been systematically linked with film transparency and mechanical stiffness.

## 2. Materials and Methods

Polymer grades were selected based on catalyst type and on previous investigations on polypropylene crystallization [23,24]. A summary of the polymer key properties is presented in Table 1. All materials were produced and provided by Borealis (Linz, Austria) as cast films of 50 µm of thickness. Two single-site catalyst homopolymer (SSC-PP) grades were selected according to the amounts of regiodefects and isotacticity, with a practically identical molecular weight distribution (MWD). The two Ziegler-Natta (ZNC-iPP) grades, a homopolymer and an ethylene-propylene random copolymer (ZNC-RACO), both possess lower isotacticity but better chain regularity, as expected for Ziegler-Natta grades. The homopolymer is a reactor grade with a broader MWD, but the ZNC-RACO is produced in a visbreaking (peroxide-induced controlled degradation) step and has a similar Mw/Mn level as the SSC-iPP grades. All used materials have an MFR at 230 °C of 8 g/10 min.

To produce the films, a pilot-scale extruder was used with a diameter of 30 mm and a L/D ratio of 30, running with a multi-purpose screw suitable for PP and PE processing and with a maximal throughput capacity of 15 kg/h. The attached cast film die has a width of 300 mm and a die gap of 0.5 mm to 1 mm. The die gap was set to 0.5 mm that resulted in a final 50 µm-thick film. Both the cooling roll (named chill-roll, CR) and the secondary roll have a width of 350 mm and a diameter of 144 mm with a temperature range that can be set from 5 °C up to 150 °C, for the CR, and from 20 °C to 150 °C, for the secondary roll, thanks to an internal water cooling system. A line speed up to 30 m/min can be achieved. The air knife, pushing the extruded melt in contact with the CR, can be adjusted in both height and length. In this study, several processing parameters have been tested. Parameter combinations were selected according to previous studies [9]: three different chill-roll temperature (*T*_chill_) levels were selected and combined with two different melt temperatures (*T*_melt_) and two different throughput or line speed (TPC) levels. In total, twelve different casted films were prepared. For all processed films, a fixed secondary roll temperature of 20 °C was used to avoid possible problems during winding. Table 2 reports all the processing variations made for cast film in this work.

**Figure 1:**
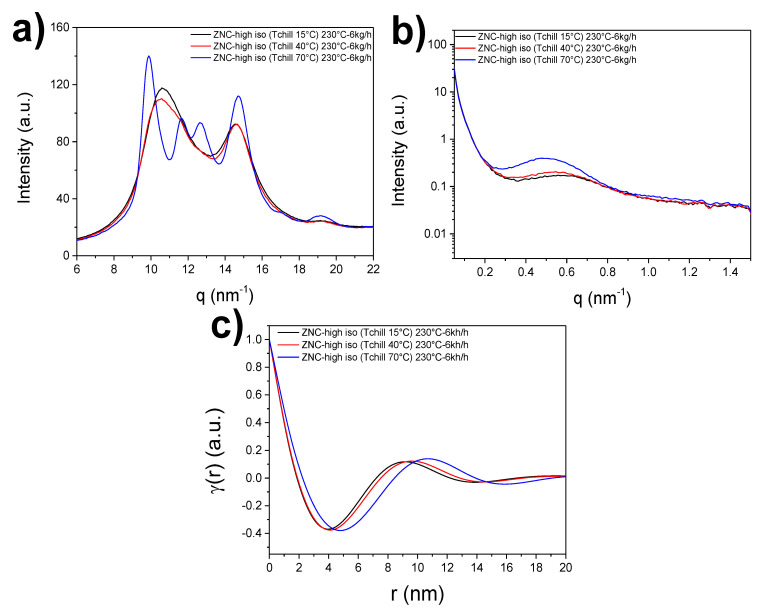
(**a**) Wide-Angle X-ray Scattering (WAXS) and (**b**) Small-Angle X-ray Scattering (SAXS) profiles for the Ziegler-Natta isotactic polypropylene (iPP) homopolymer processed at *T*_melt_ = 230 °C and TPC = 6 kg\h and with variable chill-roll temperatures, *T*_chill_; (**c**) Linear correlation function γ(r) computed for the SAXS profiles in (**b**).

The film nanostructure has been characterized in terms of internal crystalline structure, phase composition, and lamellar size via Small- and Wide-Angle X-ray Scattering (SAXS, WAXS). Figure 1 shows the WAXS and the SAXS profiles and the correlation function extracted from the SAXS data for the Ziegler-Natta iPP grade (ZNC-high-iso) processed at the three different chill-roll temperatures. Both SAXS and WAXS measurements were performed at the MINA diffractometer of the University of Groningen, equipped with a Cu rotating anode emitting X-ray wavelength at 1.5413 Å (corresponding to 8 keV). The 2D scattering patterns were collected using a Bruker Vantec 2000 2D detector for SAXS, placed at 300 cm, and a Bruker Vantec 500 2D detector for WAXS, placed at 12 cm away from the samples, respectively. Depending on the scattering power of each film, the acquisition times were changed from 30 min to 60 min in order to have very low signal-to-noise ratios and collect high quality data. The 2D patterns were converted into 1D scattering profiles using the Fit2D software (ESRF, Grenoble, France) [25], and the pixel scale was converted into the angular (2θ) and subsequently into the q scale (with q=4πsinθ/λ) using the known peak positions from a standard silver behenate sample. 

Crystalline phase composition was calculated from the WAXS profiles using a home-made written MATLAB multi-peaks deconvolution routine. For all our samples, the X-ray diffraction profiles rise from the sum of contributions from the α, the meso-, and the amorphous phases, as shown in Figure S1 of the Supplementary Materials. Each WAXS profile is reconstructed by using a linear combination of Voigt functions, in which each one of them reproduces a different scattering reflection characteristic for each phase (e.g., in Figure S1b,c, a dark green line is used to fit the (110) scattering reflection of the α form). The total simulated profile obtained by the linear combination of the Voigt functions is then used to fit the recorded intensity profile (the red line in Figure S1). The content of each phase was calculated by the ratio of the area of each Voigt peak (Ai) to the total profile area (Atot), according to Equation (1):

(1) $$Xi=\frac{Ai}{Atot}$$

By these means, the relative composition in terms of the α-monoclinic, mesomorphic, and amorphous phases can be calculated accurately [26]. The standard deviation on the relative phase composition was calculated by collecting WAXS patterns on five different spots for each cast film and by averaging the crystallinity values over the five tests. 

The long period (Lp) was calculated by data reduction of the SAXS patterns according to the so-called linear electron density correlation function, using Equation (2) [27]

(2) $$\gamma \left(r\right)=\overset{\infty }{\underset{0}{\int }}q^{2}I\left(q\right)\cos\left(qz\right)dq$$

where I(q) is the scattering intensity and r is the distance in real space.

Corresponding lamellar thickness was obtained by multiplying the extracted long period by the alpha crystallinity obtained by WAXS, as shown in Equation (3):

(3) Lc=Lp×X(α+m)

where X(α+m) is the sum of the alpha and mesophase fractional content in each sample. The standard deviation on the long period was calculated by collecting SAXS patterns on five different spots for each cast film and by averaging the long period values over the five tests after the application of the linear electron density correlation function.

The film macrostructural properties have been investigated in terms of mechanical and optical properties. Tensile moduli along the machine direction (MD) were determined according to ISO 527-3 on a Zwick Z005 U (Linz, Austria) on 15 mm wide strips at a crosshead speed of 100 mm/min and a temperature of 23 °C. Haze was determined according to ASTM D1003-0 on a Haze Gard Plus instrument. Haze is defined as the fraction of transmitted light that deviates from the incident beam by more than 2.5° [28]. In this study, no differentiation was made between surface and bulk contribution to the haze.

In order to provide the reader with all the quantitative information, all the values of the results obtained by the above mentioned analysis, and used in the figures below, are summarized in Tables S1–S4 (Supplementary Materials) for all investigated grades.

Surface investigation on some iPP cast film has been done with a DI Enviroscope AFM by Bruker. All acquisitions were performed in tapping mode at ambient temperature, with a lateral scan size of 5 µm.

## 3. Results and Discussion

### 3.1. Processing to Nanostructure

**Figure 2:**
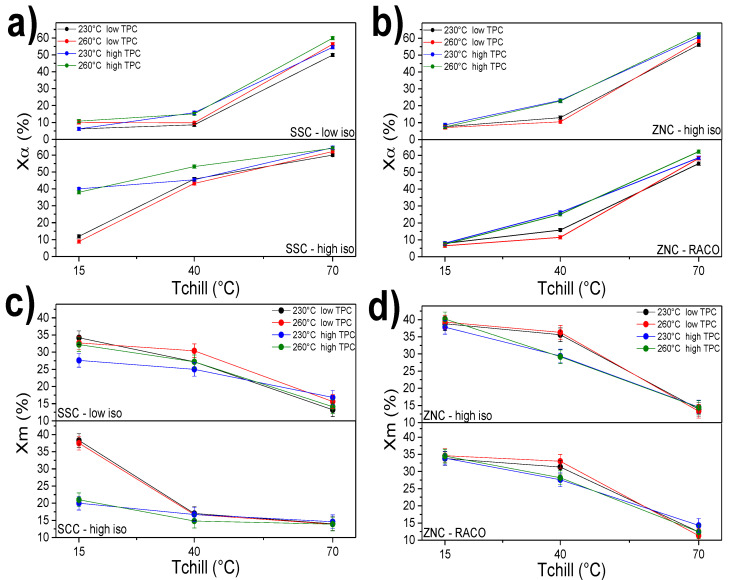
Relative α and mesophase content as a function of *T*_chill_ for all the cast film extruded polymers. (**a**,**b**) Relative α content (Xα); (**c**,**d**) relative mesophase content (Xm).

The effect on the crystalline structure and phase composition of each processing condition is discussed at first for all the studied grades. Figure 2 illustrates the fraction of the α phase and of the mesophase at the three different chill-roll temperatures, *T*_chill_. The processing parameters are pointed out in each graph’s legend. As expected, the dominant processing condition for all grades is clearly the set temperature of the first cooling roll, as this parameter is the main one responsible for the biggest change in the cooling rate applied to the polymer melt. Generally, *T*_chill_ = 70 °C allows for the development of a high amount of the monoclinic α phase in the range of 50% to 65% as the result of the lower supercooling to which the melt is subjected (Figure 2a,b). As a consequence, the formation of the mesomorphic phase occurs only in a small extent (Figure 2c,d). At this higher chill-roll temperature, the overall effect of the melt temperature and line speed on the crystallinity is rather weak, with variation in the order of a few percent for all grades. For all grades, the highest amount of α phase content (i.e., highest achieved crystallinity) is obtained when *T*_melt_ = 260 °C and combined with the highest line speed. Regardless of the iPP grade, by looking at the mesophase plot versus the chill-roll temperature of Figure 2c,d, one sees that a consistent amount of the amorphous phase is always present, in the range of 35% to a minimum of 20%, even at *T*_chill_ of 70 °C, which is the more favorable for the overall polymer crystallization. This is due to the generally very high cooling rates at which cast film products are produced and, indeed, to the particularly low chill-roll temperatures tested in this study. Similar effects were observed in previous works on cast film extrusion [9,29]. 

For *T*_chill_ = 40 °C, an interesting effect of the throughput (TPC) is observed. At this intermediate roll temperature, a wider variability of the sample crystallinity occurs by changing TPC, as shown in Figure 2. Because of the greater undercooling generated by the lower *T*_chill_, all grades exhibit consistently lower α-monoclinic phase and higher mesomorphic phase content. The melt temperature, again, has a minor effect and induces only small changes in crystallinity. On the other hand, the line speed has a more profound effect on both α and mesophase formation: samples processed at 12 kg/h have consistent higher crystallinity than those processed with 6 kg/h. The variation is particularly strong in the Ziegler-Natta grades, probably due to the combination of undercooling and high polydispersity for the ZNC-high-iso and the hinderance of the random distribution of ethylene units for the RACO grade. Under tough crystallization conditions, it is known that polymers with wide Mw/Mn and crystal defects are more easily subjected to orientational and shear effects, which can explain the particular behavior at this intermediate *T*_chill_ [7]. The two SSC- iPP grades show similar trends, but with lower magnitude compared to the two ZNC-grades. A remarkably high degree of crystallinity is shown by the more perfect single-site iPP, namely the SSC-high-iso. Here, almost 40–50% of α phase is reached at the two lower *T*_chill_ for every other condition. Orientational effects during crystallization and an overall easier crystal formation could be responsible for the very high crystallinity of this grade. The discussion for this case will be developed in a later part of this manuscript. At the lower *T*_chill_ of 15 °C, all cast films show a similar degree of crystallinity, with minor variations induced by the other two processing parameters, except for the SSC-high-iso processed with high TPC. The undercooling in this condition is enough to almost totally suppress the formation of the more stable monoclinic phase. As stated before, even if a large amount of defective, metastable mesophase is reached, about 50% of the sample is present as amorphous chains. The global undercooling effect produced by the chill-roll on the film’s total crystallinity is summarized in Figure S2 in the Supplementary Materials: it is clear that the two lowest *T*_chill_ allow for a finer tune of the material crystallinity in combination with the changes in the other machine settings. Since a way stronger effect of the chill-roll on the processing has been confirmed, we decide, from now on, to call *primary setting* the *T*_chill_ and *secondary settings* the *T*_melt_ and TPC.

**Figure 3:**
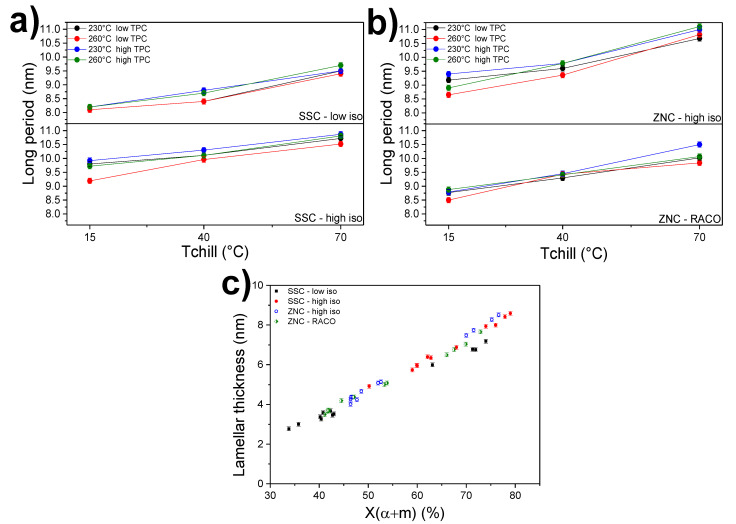
(**a**,**b**) Long period (calculated via γ(r)) as a function of *T*_chill_ for all grades; (**c**) Lamellar thickness (calculated via γ(r), Equation (2)) versus total crystallinity (WAXS) for all cast films.

To account for the effect of the processing conditions on the formation of ordered crystalline domains (namely the lamellar stacks) in the investigated iPP cast films, SAXS measurements were conducted. After reduction of the 2D SAXS patterns, the intensity plots were used to calculate the linear correlation function (see Figure 1c). The first maximum of the correlation function is assigned to the lamellae long period and calculation of the lamellar crystalline layer were done via Equation (1). Figure 3 shows the long period evolution as a function of *T*_chill_ (a) and the evolution of lamellar thickness with the total crystallinity (b) for all grades. As expected, the chill-roll temperature has a profound effect on the long period. The average long period dimension is around 9.5 nm for the more perfect grades (SSC and ZNC with high isotacticity), reaching 10.5–11 nm at *T*_chill_ = 70 °C. In accordance with the WAXS results, the single-site iPP with a lower tacticity and a higher amount of regiodefects, SSC-low-iso, shows the thinnest crystalline layers, even below the values found for the random copolymer (see values in Table S1). Chain defects produced by non-optimal monomer insertion, typical for SSC-iPP grades, cause a higher reduction on the crystal’s growth size, eventually yielding smaller lamellae, in comparison to the inclusion of comonomers in the crystal structure. This result is in agreement with previous investigations on single-site grades [2,6]. Again, the SSC-iPP with better regio- and stereoregularity (SSC-high-iso) exhibits the highest lamellar period and largest thickness (Figure 3a,c respectively) at all chill-roll temperatures.

For all grades, the effect of the secondary parameters is quite small. Overall, an orientational effect could be present for all polymers, especially at the higher *T*_chill_ of 70 °C because of the similar α-crystallinity. In Figure 3, the lamellar thickness is plotted against the total crystallinity for all investigated cast films. The evolution is clearly linear for all iPP and the two with better overall regularity (SSC- and ZNC-high-iso) stand with larger crystalline layers. Samples with a total crystallinity in the range of 30% to 40% are mostly mesomorphic, quenched at a chill-roll temperature of 15 °C. The crystal size calculated for these samples is around 2.5–2.8 nm and it is close to the minimum crystal size reported for mesomorphic iPP [30]. In order to better understand the possible presence of orientational effect in the produced cast film, azimuthal integration of the WAXS signals was performed. Figure S3 shows the intensity profiles versus the azimuthal angle (360°) for all investigated samples processed at the lower *T*_melt_ (230 °C). It is clear that a significant orientation is present at the highest *T*_chill_ used, at 70 °C. Only for the more perfect SSC-high-iso (Figure S3b), a clear orientation is present at the two lowest *T*_chill._ Overall, these data seem to exclude the possibility of a high flow-induced crystallization phenomenon.

### 3.2. Nano-Structure to Macro-Properties 

**Figure 4:**
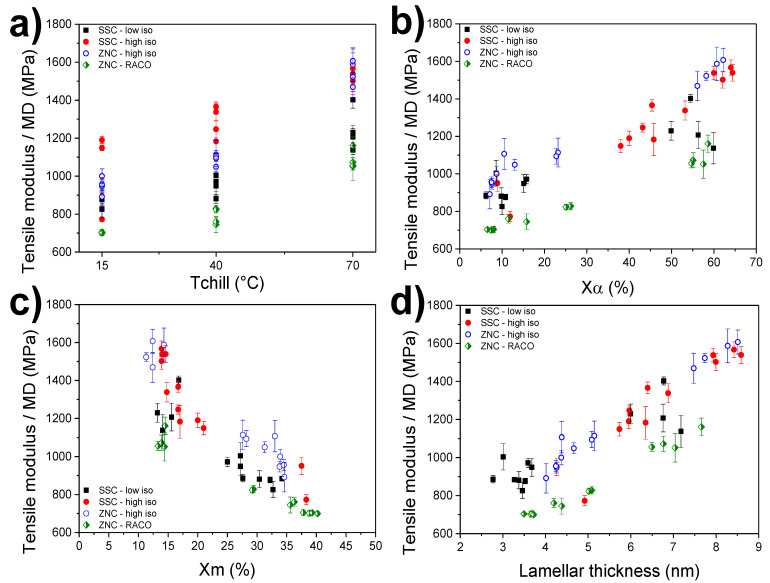
Tensile modulus along the machine direction as a function of (**a**) the chill-roll temperature; (**b**) the relative α phase content; (**c**) the relative mesophase content and (**d**) the lamellar thickness, for all grades and processing conditions.

After an evaluation of the effects that processing conditions have on the nanostructure of each cast film, the consequent effect on the macroscopic mechanical and optical properties has been investigated. In Figure 4a, the tensile modulus along the machine direction is plotted against the chill-roll temperature for all investigated cast films. As expected, and following the WAXS results, the effect of this primary machine setting is rather strong for all polymer types. In a first approximation, the trend seems to be only determined by the film crystallinity, with the highest *T*_chill_ yielding higher stiffness overall in the range of 1400 MPa to 1600 MPa. The two lower chill-roll temperatures, 15 °C and 40 °C, show a consistent reduction on the modulus, which is in line with the overall lower values of crystallinity observed by WAXS. However, differences in mechanical properties are clearly appreciable between the different grades, highlighting the effect of the polymer’s structural composition. For the random ethylene-propylene copolymer, the hindrance of the crystallization caused by the inclusion of the C2 units yields more ductile behavior at all chill-roll conditions. A similar behavior is observed for the more stereo-defective single-site sample (SSC-low-iso), which shows a linear drop in tensile modulus when *T*_chill_ decreases. In this case, the different effect of stereo and regio defects seems to be of similar magnitude and thus it is not easy to distinguish the effect of the secondary machine settings on the two grades. Again, the use of a higher line speed and a higher melt temperature results in films with a slightly higher modulus at any given *T*_chill._ The mechanical properties of all cast films have been subsequently confronted with the content of the α and mesomorphic phases, as shown in Figure 4b,c. Even with some level of data-scattering, a rather linear relationship between the modulus and the α-crystallinity is observed. The two more perfect iPPs (SSC- and ZNC-high-iso) show overall the best mechanical properties at every degree of crystallinity. As expected from previous results (Figure 2a), the high α-crystallinity of the SSC-high-iso films quenched at 15 °C yield moduli values in the range of 1100–1300 MPa that are close to those of films processed with the two higher *T*_chill_ of 15 °C and 70 °C. The presence of ethylene comonomer, however, yields a more ductile material when compared with all other grades, even at high crystallinity (around 60%). Figure 4c shows the strong effect of the mesophase presence on the mechanical properties of iPP materials. An increase in the mesophase content, mostly driven by the lowering of the chill-roll temperature, produces more ductile film for all investigated grades. Again, the more perfect SSC-high-iso and ZNC-high-iso grades present the highest moduli at all conditions. 

In order to take the effect of the line speed on both crystallinity and mechanical properties into account, a correlation with lamellar thickness is needed. Previous work on the relation between the crystalline structure and mechanical properties of iPP suggests that for a given degree of crystallinity, polymers with higher chain regularity will present higher lamellar thickness, yielding a stiffer material [31]. Figure 4d shows the moduli plots versus the lamellar thicknesses for all batches. The resemblance to the behavior found for the α-crystallinity is clear. Interestingly, it is found that the effect of the line speed (12 kg/h vs. 6 kg/h) on the mechanical properties could be an orientational one, since cast films with similar crystallinities also have similar lamellar thicknesses. This effect can be easily confirmed in the cast film with overall α-crystallinity above 40% and for the two SSC grades. However, why the line speed effect is stronger for the polymer with lower polydispersity (SSC-low-iso) is unclear, since higher polydispersity is known to enhance crystallization under shear conditions [7]. 

**Figure 5:**
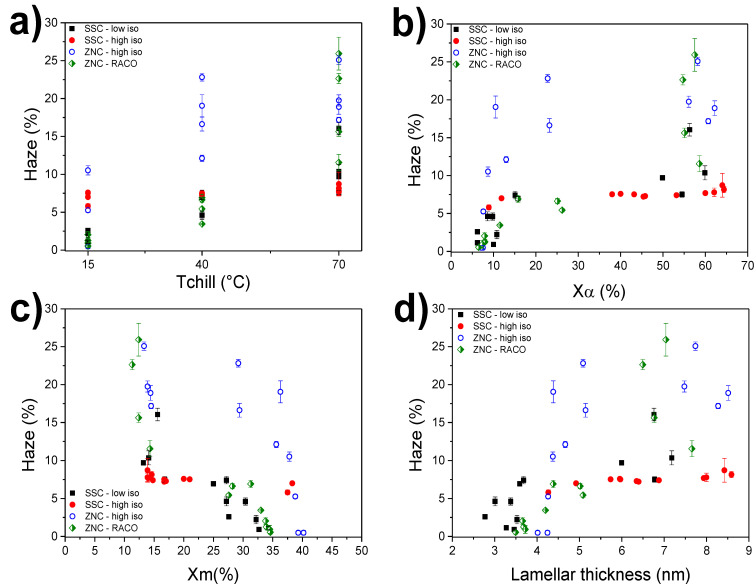
Haze as a function of (**a**) chill-roll temperature; (**b**) α-crystallinity; (**c**) mesophase content; (**d**) lamellar thickness, for all investigated grades.

The optical performance of the cast film extruded polymers has been evaluated by means of haze measurements. In cast film technology, production of highly transparent materials is of mandatory importance since packaging application requires, along with good mechanical properties, outstanding light transmittance and product visibility. In Figure 5a–d, the recorded haze values for all grades have been correlated to the primary machine settings (*T*_chill_), the X-ray crystallinity, and the lamellar thickness to give a better understanding of the effect of the processing condition on the final optical performance. Firstly, the direct effect of the undercooling is shown in Figure 5a. Films cooled at the lowest temperature (*T*_chill_ = 15 °C) present the best haze values (all below 10%) if compared to those cooled at the higher *T*_chill_. This result is in line with the trends of the film crystallinity (Figure 5b) since the presence of well-developed lamellar domains of α-monoclinic crystals causes the incident light to be more scattered in comparison to the mesomorphic domains. The nodular morphology typical for the defective mesomorphic iPP phase has a size of 5–20 nm, which accounts for very low haze values while ensuring good flexibility and ductility. The two single-site grades reach better transparency levels, mostly at high chill-roll temperatures, compared to the Ziegler-Natta ones. This is probably due to the narrower molecular weight distribution of the two SSC-iPPs, which leads to better crystal homogeneity. In addition, the presence of ethylene comonomers in the RACO sample are known to promote lower haze, because of the crystallization hindrance caused by the foreign monomeric units. An interesting behavior is shown by the SSC-high-iso films, which exhibit a quite constant haze level (around 6–9%) regardless of the processing conditions. This issue has been previously reported [9,32] and a clear correlation between haze and α-crystallinity for this sample is not easy. In our study, a better correlation between haze and mesophase content is observed (Figure 5c). Sharp haze changes are observed at around 15% and at 30–35% of mesophase content for all grades, with the two SSC and the RACO films having better transparency. For the more perfect ZNC grade, a higher amount of mesophase is needed to reach good transparency levels (about 40%). As discussed above, this two-step behavior is not observed for the SSC-high-iso that exhibits a rather stable haze level. 

The line speed (TPC) has a more pronounced effect on the optical properties compared to the mechanical properties. This secondary setting produces stronger variations, especially for the Ziegler-Natta iPP. Furthermore, we attempted to correlate the haze values with the calculated lamellar thickness for all grades (Figure 5d). Except for the case of the SSC-high-iso, the smaller the crystal size, the higher the transparency. For the SSC-high-iso, a high nucleation density combined with the high isotacticity gives smaller crystalline domains that can lead to a smoother surface and thus better crystallinity [33]. This consideration can be confirmed by Figure S4, which shows an AFM height comparison between the SSC-high-iso and a ZNC-high-iso at two *T*_chill_ of 15 °C and 70 °C at the same secondary settings (230 °C, 12 kg/h). Clearly, smaller globular structures are present in the SSC-high-iso at both cooling temperatures compared to the Ziegler-Natta grade. The high polydispersity of the ZNC-high-iso is again accountable for the high haze level even at the lower *T*_chill_ of 15 °C and 40 °C. It is known that contributions to haze arise from both superficial and bulk structure [13,33]. Recently, α crystals located at the film surface and contributing to the increase of the surface roughness have been reported for cast film extruded iPP [9]. An investigation of the surface and bulk effects in iPP cast film products via surface-sensitive X-ray methods is ongoing and will be published in the future.

## 4. Conclusions

A quantitative investigation on isotactic polypropylene materials of different chain structures resulting from catalyst nature and copolymerization produced by cast film extrusion technology has been performed, in order to elucidate the effects that various processing settings have on the optical and mechanical performance of the film. Here, the temperature of the melt, the temperature of the chill-roll, and the line speed have been varied systematically. It is found that the dominant (or primary) machine parameter is the temperature of the cooling roll, which determines the undercooling to which the polymer melt is subjected. A lower chill-roll temperature of 15 °C produced films with a high content of mesomorphic phase, eventually giving ductile and transparent films. The intermediate and high chill-roll temperature, respectively 40 °C and 70 °C, mostly produced α-monoclinic materials with better stiffness and a generally worse level of transparency. It was found that the secondary processing parameters, such as the melt temperature and line speed, influence the film crystallinity (and hence the properties) mainly at the intermediate chill-roll temperature. The line speed has been found to produce a stronger effect on the mechanical and optical properties and an unexpected orientational effect is present, as similar levels of crystallinity and lamellar thickness are often reached. The effects of the high polydispersity and presence of comonomers of the two Ziegler-Natta catalyzed polymers are in line with previous studies on polymer crystallization under industrial conditions. On the other side, single-site catalyst grades yield films with overall better optical performances and lower stiffness. Interestingly, the single-site catalyst iPP with high isotacticity shows very low haze values even at the highest crystallinity level, while the haze values for the iPP Ziegler-Natta homopolymer grade are the highest ones observed. Although a clear relationship between catalyst nature, polymer characteristics, and machine settings has been made, further investigations into the surface and bulk contributions towards transparency levels are needed in the cast film technology field. Our future works will focus on determining the precise contributions of surface roughness and bulk composition on the optical performance of such important industrial products.

## Figures and Tables

**Table 1:** Overview of the polymer characteristics for the grades used in this work.

Polymer	*T*_melt_ (°C)	Isotacticity <mm> (%)	2,1 Regiodefects (%)	*M*_w_/*M*_n_	C2 (wt %)
SSC–low-iso	154	99.2	0.70	3.5	0
SSC–high-iso	160	99.8	0.23	3.5	0
ZNC–high-iso	165	98.9	0.0	5.4	0
ZNC–RACO	153	96.5	0.0	3.4	2.2

**Table 2:** Overview of the processing parameter variations in the cast film production of this study.

Processing Parameters			
Chill-roll temperature (*T*_chill_—°C)	15	40	70
Melt temperature (*T*_melt_—°C)	230	260	
Throughput capacity (TPC—kg\h)	6	12	

## Supplementary Materials

The following are available online at https://www.mdpi.com/2073-4360/12/8/1636/s1, Figure S1: Fitting of the WAXS profiles for the ZNC-high-iso grade processed at *T*_melt_ 230 °C, TPC 6 kg\h, and at different *T*_chill_: a) 15 °C; b) 40 °C; c) 70 °C. Figure S2: Total crystallinity (α and mesomorphic) versus *T*_chill_ for all investigated films. Figure S3: WAXS azimuthal integration. Figure S4: AFM height images at 5µm scale for a) SSC-high-iso; b) ZNC-high-iso. Table S1: Results overview for iPP single-site catalyst homopolymer SSC-low-iso. Table S2: Results overview for iPP single-site catalyst homopolymer SSC-high-iso. Table S3: Results overview for iPP Ziegler-Natta catalyst homopolymer ZNC-high-iso. Table S4: Results overview for iPP Ziegler-Natta catalyst random copolymer ZNC-RACO.
