# Supplementary material for: Systematic Investigation on the Structure-Property Relationship in Isotactic Polypropylene Films Processed via Cast Film Extrusion
Source: Polymers (Basel). 2020 Jul 23;12(8):1636. doi: 10.3390/polym12081636 (PMC7464016; doi:10.3390/polym12081636)
Supplement: Supplementary file 1 [file polymers-12-01636-s001.pdf]

Supporting Information

Systematic Investigation on the Structure-Property Relationship in Isotactic Polypropylene Films Processed via Cast Film Extrusion

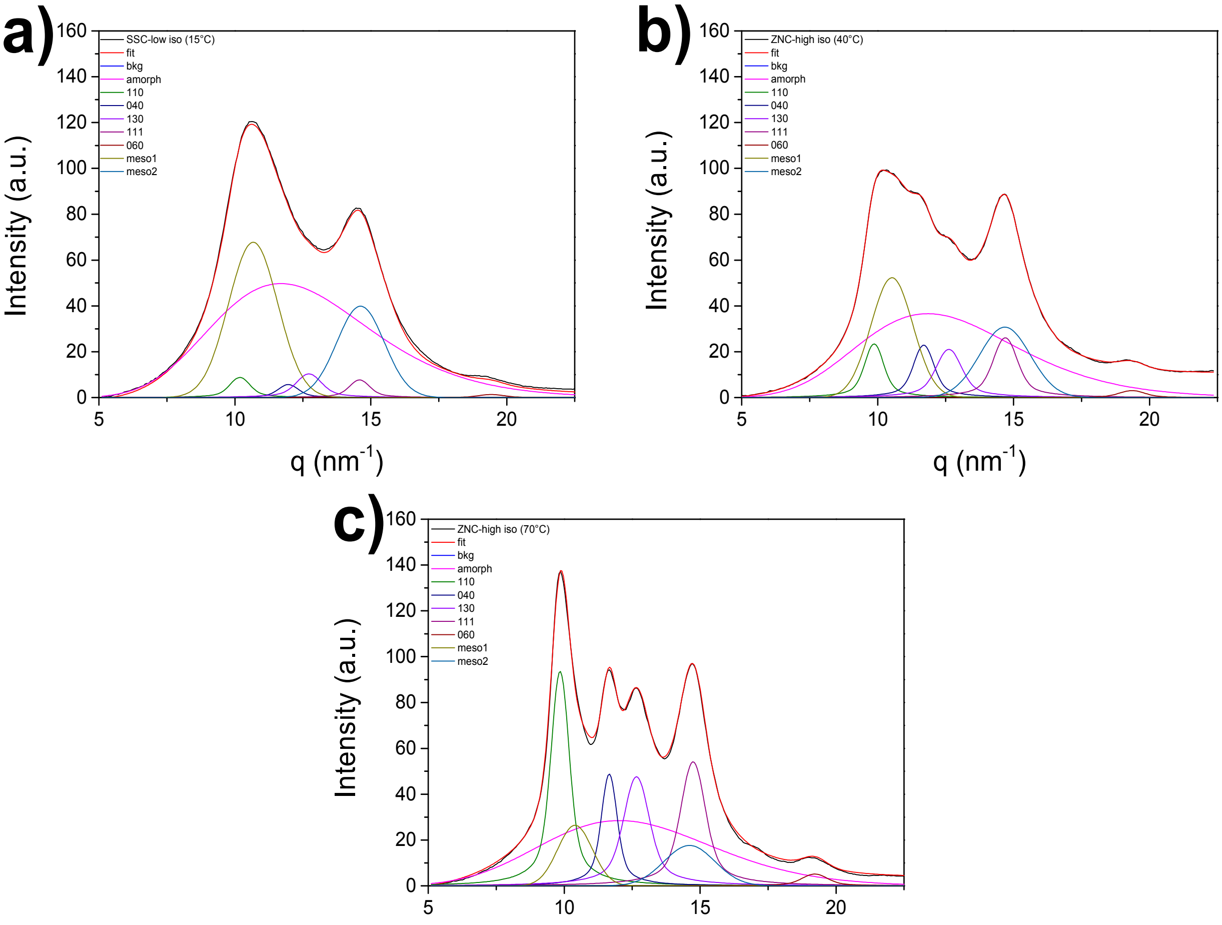

Figure S1: Fitting of the WAXS profiles for the ZNC-high iso grade processed at T_{melt} 230°C, TPC 6 kg\h and at different T_{chill} : a) 15°C; b) 40°C; c) 70°C

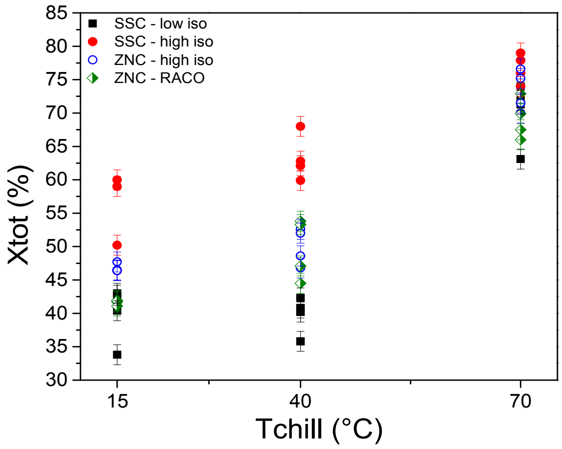

Figure S2: Total crystallinity (α and mesomorphic) versus T_{chill} for all investigated films

Table S1: Results overview for iPP Single Site Catalyst homopolymer SSC-low-iso

SSC – low iso	T_{melt} (°C)	T_{chill} (°C)	TPC (kg/h)	Xα (%)	Xm (%)	Xamorph (%)	TM (MD) (MPa)	Haze (%)	Long period (nm)	Lamellar thickness (nm)
CV1	230	15	6	6,2	34,2	59,6	884	1,13	8,1	3,27
CV2	230	15	12	6,2	27,6	66,2	886	2,6	8,2	2,77
CV3	230	40	12	15,8	25,0	59,2	972	6,95	8,8	3,59
CV4	230	40	6	8,6	27,2	64,2	1004	4,62	8,4	3,01
CV5	230	70	6	49,9	13,2	36,9	1230	9,71	9,5	5,99
CV6	230	70	12	54,5	16,8	28,7	1402	7,51	9,5	6,77
CV7	260	15	12	10,8	32,2	57,0	877	2,22	8,2	3,53
CV8	260	15	6	10,0	32,7	57,3	826	0,91	8,1	3,46
CV9	260	40	6	9,8	30,4	59,8	881	4,6	8,4	3,38
CV10	260	40	12	15,1	27,2	57,7	949	7,42	8,7	3,68
CV11	260	70	12	59,9	14,1	26,0	1137	10,36	9,7	7,18
CV12	260	70	6	56,3	15,6	28,1	1207	16,05	9,4	6,76

Table S2: Results overview for iPP Single Site Catalyst homopolymer SSC- high-iso

**SSC – high iso**	**T_{melt} (°C)**	**T_{chill} (°C)**	**TPC (kg/h)**	**Xα (%)**	**Xm (%)**	**Xamorph (%)**	**TM (MD) (MPa)**	**Haze (%)**	**Long period (nm)**	**Lamellar thickness (nm)**
CV13	230	15	6	11,9	38,3	49,8	773	7	9,8	4.92
CV14	230	15	12	40,0	20,0	40,0	1190	7,59	9,9	5.95
CV15	230	40	12	45,4	16,7	37,9	1366	7,22	10,3	6.40
CV16	230	40	6	45,8	17,0	37,2	1183	7,29	10,1	6.35
CV17	230	70	6	60,0	14,0	26,0	1538	7,69	10,7	7.93
CV18	230	70	12	64,4	14,6	21,0	1539	8,16	10,9	8.59
CV19	260	15	12	38,0	21,0	41,0	1149	7,53	9,7	5.73
CV20	260	15	6	8,9	37,5	53,6	951	5,8	9,2	4.26
CV21	260	40	6	43,2	16,7	40,1	1247	7,52	10,0	5.97
CV22	260	40	12	53,2	14,8	32,0	1338	7,41	10,1	6.87
CV23	260	70	12	64,0	13,9	22,1	1567	8,72	10,8	8.42
CV24	260	70	6	62,1	13,9	24,0	1502	7,79	10,5	8.00

Table S3: Results overview for iPP Ziegler-Natta Catalyst homopolymer ZNC-high-iso

ZNC – high iso	T_{melt} (°C)	T_{chill} (°C)	TPC (kg/h)	Xα (%)	Xm (%)	Xamorph (%)	TM (MD) (MPa)	Haze (%)	Long period (nm)	Lamellar thickness (nm)
CV1	230	15	6	7,6	38,8	53,6	947	5,27	9,2	4,26
CV2	230	15	12	8,7	37,8	53,5	1000	10,53	9,4	4,37
CV3	230	40	12	23,2	29,4	47,4	1114	16,62	9,8	5,14
CV4	230	40	6	13	35,6	51,4	1049	12,12	9,6	4,67
CV5	230	70	6	56,1	13,9	30,0	1469	19,75	10,7	7,48
CV6	230	70	12	60,7	14,5	24,8	1587	17,18	11,0	8,27
CV7	260	15	12	7,5	40,2	52,3	956	0,48	8,9	4,25
CV8	260	15	6	7,1	39,3	53,6	892	0,48	8,7	4,01
CV9	260	40	6	10,5	36,3	53,2	1107	19,05	9,4	4,38
CV10	260	40	12	22,8	29,2	48,0	1094	22,82	9,8	5,09
CV11	260	70	12	62,2	14,4	23,4	1607	18,9	11,1	8,51
CV12	260	70	6	58,2	13,3	28,5	1523	25,08	10,8	7,74

Table S4: Results overview for iPP Ziegler-Natta Catalyst random copolymer ZNC-RACO

ZNC – RACO	T_{melt} (°C)	T_{chill} (°C)	TPC (kg/h)	Xα (%)	Xm (%)	Xamorph (%)	TM (MD) (MPa)	Haze (%)	Long period (nm)	Lamellar thickness (nm)
CV13	230	15	6	7,9	33,8	58,3	702	2,04	8,8	3.66
CV14	230	15	12	8,0	33,9	58,1	705	1,26	8,8	3.68
CV15	230	40	12	26,2	27,6	46,2	828	5,44	9,5	5.08
CV16	230	40	6	15,8	31,3	52,9	745	6,92	9,3	4.38

CV17	230	70	6	55,1	12,4	32,5	1073	15,63	10,0	6.76
CV18	230	70	12	58,6	14,3	27,1	1161	11,57	10,5	7.65
CV19	260	15	12	7,4	34,5	58,1	700	0,93	8,9	3.72
CV20	260	15	6	6,5	34,6	58,9	704	0,54	8,5	3.49
CV21	260	40	6	11,5	33,0	55,5	761	3,45	9,4	4.20
CV22	260	40	12	25,1	28,2	46,7	823	6,63	9,4	5.02
CV23	260	70	12	57,5	12,4	30,1	1052	25,93	10,1	7.04
CV24	260	70	6	54,7	11,3	34,0	1056	22,63	9,8	6.49

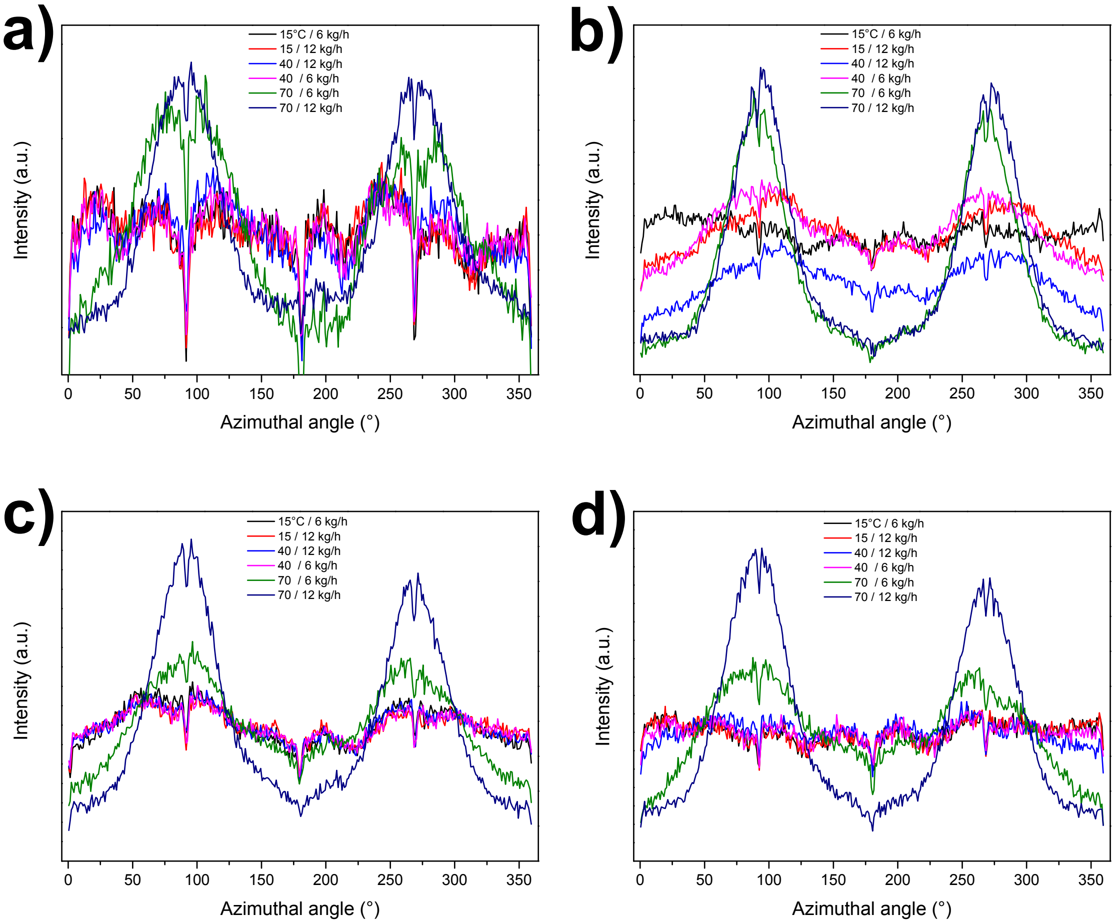

Figure S3: WAXS azimuthal integration for a) SSC-low-iso; b) SSC-high-iso; c) ZNC-high-iso and d) ZNC-RACO

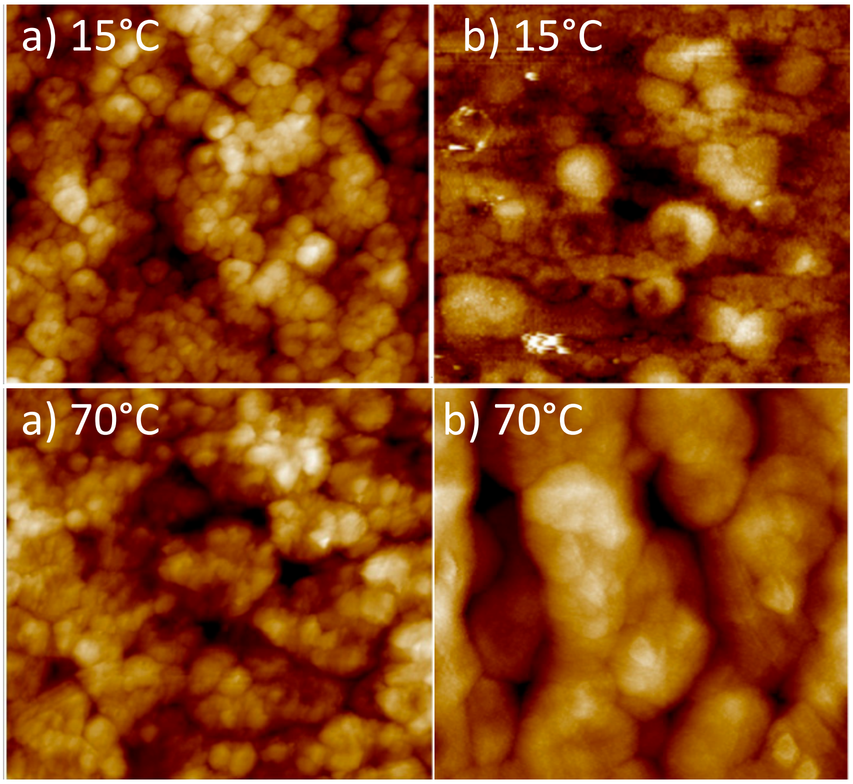

Figure S4: AFM height images at 5 µm scale for a) SSC-high-iso; b) ZNC-high-iso at two different T_{chill}
